# Mitigating Psychological Symptoms in Public Safety Personnel Through Supportive Text Messaging Program

**DOI:** 10.1192/j.eurpsy.2024.1147

**Published:** 2024-08-27

**Authors:** G. Obuobi-Donkor, R. Shalaby, E. Eboreime, B. Agyapong, R. D. L. Dias, V. Agyapong

**Affiliations:** ^1^Department of Psychiatry, Dalhousie University, Halifax; ^2^Department of Psychiatry, University of Alberta, Edmonton, Canada

## Abstract

**Introduction:**

Public safety personnel (PSPs) often suffer from mental health issues due to the challenging and intricate nature of their work. Various barriers may prevent them from seeking necessary support and treatment. Therefore, implementing innovative and cost-effective interventions can potentially enhance the mental well-being of PSPs.

**Objectives:**

The study sought to assess the influence of the Text4PTSI program on symptoms of depression, anxiety, trauma, and stress, as well as the resilience of public safety personnel after six months of receiving supportive text message intervention.

**Methods:**

PSP subscribed to the Text4PTSI program and received daily supportive l SMS text messages for six months. Participants were invited to complete standardized self-rated web-based questionnaires to assess depression, anxiety, posttraumatic stress disorder (PTSD), and resilience symptoms measured on the Patient Health Questionnaire-9 (PHQ-9), Generalized Anxiety Disorder-7 scale (GAD-7), Posttraumatic Stress Disorder Checklist-Civilian Version (PCL-C), and the Brief Resilience Scale (BRS), respectively. The assessment of mental health conditions was conducted at enrolment, six weeks, three months, and six months after enrollment.

**Results:**

One hundred and thirty-one subscribers participated in the Text4PTSI program. A total of 31 participants completed the baseline survey, and 107 total surveys were recorded at all follow-up time points. The baseline prevalence of likely major depressive disorder (MDD) was 47.1%, likely generalized anxiety disorder (GAD) was 37.5%, low resilience was 22.2%, and likely PTSD was 13.3%. At six months post-intervention, the prevalence of psychological conditions. There was a decrease in the mean scores on the PHQ-9, GAD-7, PCL-C, and the BRS from baseline to post-intervention by 25.8%, 24.7%, 9.5%, and 0.3%, respectively. However, the decrease was only statistically significant for the mean change in GAD-7 scores with a low effect size (t (15) = 2.73, p = 0.02).

**Conclusions:**

The results of this study suggest a reduction in the prevalence of likely MDD as well as the severity of anxiety symptoms from baseline to post-intervention for subscribers of the Text4PTSI program. The program has the potential to complement existing services, aiding in mental health support for public safety personnel.

**Disclosure of Interest:**

None Declared

